# Application of Thermosonication in Red Pitaya Juice Processing: Impacts on Native Microbiota and Quality Properties during Storage

**DOI:** 10.3390/foods10051041

**Published:** 2021-05-10

**Authors:** Wenxian Zhu, Yana Ai, Fang Fang, Hongmei Liao

**Affiliations:** 1 School of Food Science and Technology, Collaborative Innovation Center of Food Safety and Quality Control in Jiangsu Province, Jiangnan University, Wuxi 214122, China; zwx15761636900@163.com (W.Z.); 6190112001@stu.jiangnan.edu.cn (Y.A.); 2 Whistler Center for Carbohydrate Research, Department of Food Science, Purdue University, West Lafayette, IN 47906, USA; ffang@purdue.edu

**Keywords:** native microbiota, physico-chemical quality, red pitaya juice, storage, thermosonication

## Abstract

The effects of thermosonication (TS) on microbial safety and quality of red pitaya juice during storage were assessed in this study. Freshly prepared red pitaya juices were thermosonicated at 475 W and 56 °C for 20 min. Upon TS processing, native microbiota including aerobic bacteria, yeasts, and molds reduced to less than 10 CFU/mL. Their growth during storage were slow and equal to thermal-processed (83 °C, 1.5 min) samples. During storage at 4 °C for 28 days, soluble solid content, pH, activities of polyphenol oxidase and peroxidase, and browning degree remained unchanged. A visible color decay was observed in TS-processed samples at day 10, mainly resulting from decomposition of betacyanins and the growth of residual native microbiota. Compared to thermal-treated juices, better color retention was obtained by TS treatment. Therefore, TS is a promising alternative technology of thermal methods of juice processing, with equal shelf life and better quality retention effects.

## 1. Introduction

Fruit juice is popular all around the world as a good source of sugars, vitamins, polyphenols, flavonoids, and minerals. Processing fresh fruits to juices normally involves thermal treatments to inactivate microorganisms and enzymes, which avoid putrefying of fresh fruit during transportation and storage to ensure food safety and extend shelf life of fruit juice products [1]. However, high temperature treatment usually causes undesirable changes in sensory attributes, including texture, flavor, color, smell, and losses in nutritional qualities [2]. As consumers’ preference shifts towards high-quality and less or non-processed fruit juices, developing novel technologies as an alternative of thermal processing is of growing interest, especially for fruit juice processing.

Red pitaya is a kind of tropical fruit with a bright purple-red color, which is one of the attributes to attract consumers and derives from betacyanins, an antioxidant with known health benefits [3]. Recently, it had been reported that pitaya fruit were processed into juice, puree, and natural pigment extractive [4,5,6]. Proper processing of this fruit overcomes its seasonal and regional characteristics and produces high-quality products that is always in demand. Red pitaya juice attracts increasing attention due to its nutritional functionalities, special purple-red color, and soft, bright, and fresh taste. To retain the desirable characteristics of red pitaya during juice processing and storage, well-controlled processing and storage conditions should be investigated. More exactly, its characteristics of rich in natural soluble solid content (SSC) might result in that juice is susceptible to microbial spoilage and easy to decay, and its color is sensitive to heat and light. Those should be considered primarily during processing and following shelf-life estimation. Recently, some non-thermal technologies or their combination forms have been applied in pitaya juice processing, including UV-C irradiation and ozone-HHP [4,7,8]. For instance, pitaya juice subjected to ozone for 7 min and followed by high hydrostatic pressure (HHP) at 316 MPa for 5 min shows negligible color change, highest sensory preference (79%), and non-detectable levels of native microbiota after stored for 35 days at 5 ± 2 °C [4]. However, an unstable shelf life of juice that is caused by endogenous enzymes due to relatively low inactivation efficiency of these non-thermal technologies and high investment or operational costs would obstacle their industrial application for red pitaya juice processing.

Thermosonication (TS) is an energy-efficient technology by combining ultrasonication with moderate heat. The physicochemical and thermal effects formed by acoustic cavitation and ultrasound waves result in the inactivation of microorganisms and/or endogenous enzymes in food [9]. Recently, it attracted growing research interests. It has been reported that enzymes in apple juice, including polyphenol oxidase, peroxidase, and pectin methylesterase, were effectively inactivated by this technology [9]. Besides that, yeast and molds (Y&M) reduced to a level below the detection limit of plate counting assay in the same study. Compared to high temperature thermal treatment, higher contents of natural pigments, polyphenol, and flavonoids (e.g., anthocyanin) were retained in carrot juice and bayberry juice after TS treatment [10,11]. During storage at 4 °C, TS-treated juices (e.g., carrot and orange juices) had a slow growth rate of total mesophiles and Y&M [12,13], suggesting its potential for red pitaya juice processing. However, there is a knowledge gap on tracing quality index, nutritive value, and microbial safety of TS-processed juices during storage, they are the basic guarantee for evaluating and predicting their shelf lives.

Our previous study revealed that TS preserves the color of red pitaya juice better than thermal treatment, but still results in degradation and isomerization of betacyanins, as well as non-enzymatic browning [14]. Color is an important attribute relating with the visual appeal and quality of fruit juice [15]. Generally, color changes of fruit juice are mainly related to enzymatic browning of phenolic compounds, degradation of pigments, and non-enzymatic browning during or after processing [16]. Nevertheless, no literature has reported physico-chemical and microbial qualities of clear red pitaya juice treated by TS during storage. It is important and the premise of its application in industrial scale. Therefore, the objectives of this study were to evaluate the microbial safety and quality index of TS-processed clear red pitaya juice during storage, and to assess the possibility of TS as an alternative technology of thermal processing.

## 2. Material and Methods

### 2.1. Preparation of Clear Red Pitaya Juice

Preparation of clear juice of red pitaya fruit (*Hylocereus polyrhizus*) followed the method as described by Liao et al. [14]. Two batches of clear red pitaya juice, 12 L of each batch, with initial soluble solid content of 12.9 ± 0.1 ^o^Brix and pH = 4.77 ± 0.01, were prepared in December 2018 and June 2019, respectively. Obtained juice was stored in sterilized glass bottles at 4 °C until further treatments.

### 2.2. Experimental Design

**Figure 1:**
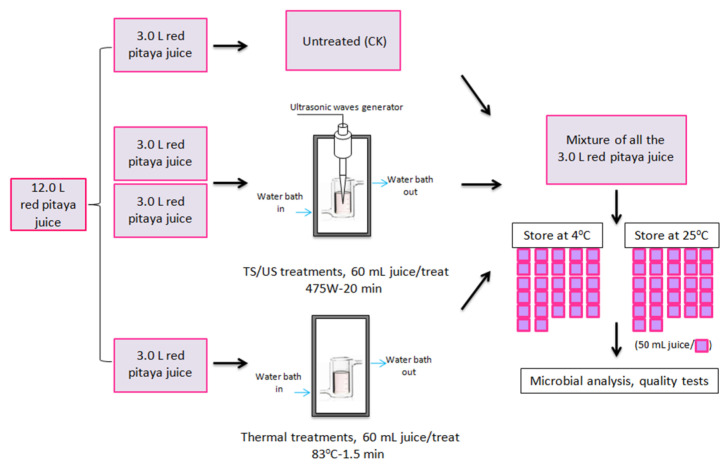
A schema of the experimental design for each batch.

The above-mentioned two batches of juice were divided into four groups randomly, to be treated by TS, ultrasonication (US), and thermal processing, with a group untreated as control samples (Figure 1). The untreated and treated red pitaya juice samples in glass bottles (50 mL/bottle) were stored at 4 ± 1 °C and 25 ± 1 °C, respectively; they were sampled every other day at 25 °C, and were sampled on days 2, 4, 7, 10, 15, 20, 25 and 28 during storage at 4 °C. The microbial and quality index of those samples were analyzed immediately. All treatments were repeated at least twice. The analytical measurements on pitaya juices for each treatment were performed in triplicate.

### 2.3. Thermosonication, Ultrasonication, and Thermal Treatments

As described in Liao et al. [17], TS treatments were conducted using an ultrasonic processor with 10 mm probe (Scientz-IID, Ningbo Scientz Biotechnology Co., Ltd., Zhejiang, China). Each 60 mL clear red pitaya juice in 150 mL double-walled glass vessel was adjusted to 56 °C using a thermostatically controlled water bath (DC0506, Hengping Instrument & Meter Factory, Shanghai, China). The input power was 475 W, the pulse time interval was 3 s, and the duration was 20 min. US treatments were conducted using the above-mentioned ultrasonic processor, with samples’ temperature controlled at 10 ± 1 °C. Thermal treatments were conducted as following: 60 mL juice in a 150 mL double-walled glass vessel was connected with a temperature-controlled water bath and held for 1.5 min since its center temperature reached 83 °C. Samples were incubated in an ice-water bath immediately after treatments until further analysis.

### 2.4. Microbial Analysis and Quality Assay

#### 2.4.1. Microbial Enumeration

The detection of viable total aerobic bacteria (AB) and Y&M in clear red pitaya juice followed the method described by Liao et al. [17]. Samples were serially diluted at 10 times gradient with 0.85% sterile saline solution, and 1 mL of each dilution was spread on duplicate sterile petri dishes containing the Nutrient agar (Sinopharm Chemical Reagent Co., Ltd., Shanghai, China) and the Rose bengal agar (Sinopharm Chemical Reagent Co., Ltd., Shanghai, China), to enumerate residual cultivable AB and Y&M cells, respectively. The numbers of AB and Y&M were counted after incubation at 37 ± 1 °C for 48 ± 2 h, and at 28 ± 1 °C for 72 ± 2 h. The limit of plate counting detection is 1 CFU/mL.

#### 2.4.2. pH and Soluble Solid Content

The pH of juice samples was detected using a pH meter (DELTA 320, Mettler-Toledo, Zurich, Switzerland) by immersing electrode in clear red pitaya juice samples at 25 °C. The SSC was measured using a pocket refractometer (PAL-α, Atago, Tokyo, Japan) by dropping juice drops on the mirror face at 25 °C.

#### 2.4.3. Color Indexes

Color indexes of juice samples were determined according to Liao et al. [14]. Δ*E* was calculated according to Equation (1):

(1) $$\Delta E=\sqrt{{\left(L_{0}^{*}-L^{*}\right)}^{2}+{\left(a_{0}^{*}-a^{*}\right)}^{2}+{\left(b_{0}^{*}-b^{*}\right)}^{2}}$$

where L_0_*, a_0_* and b_0_* represent the values of untreated sample, L*, a* and b* represent the values of treated samples and during storage.

#### 2.4.4. Betacyanins Content

The content of betacyanins in the clear red pitaya juice was determined using a UV-visible spectrophotometer (Shimazu, UV mini-1240, Kyoto, Japan) according to the method of Marszałek et al. [18] with slight modifications. The content of betacyanins was calculated using the Equation (2):

Betacyanins content (mg/100 mL) = (*A* × *DF* × *MW* × 100)/(*ε* × *L*) (2)

where *A* is absorbance at 538 nm, *DF* is dilution factor, and *L* is path length (=1 cm), molecular weight (*MW*) = 550 g/mol, and molar extinction coefficient (*ε*) = 65000.

#### 2.4.5. Enzymes Activity

The polyphenol oxidase (PPO) activity was determined according to the method reported by Yi et al. [19] with slight modifications. Each 1 mL juice was mixed with 2 mL substrate containing 0.07 M catechol and 0.2 M phosphate buffer (pH 6.5) as the enzymatic reaction solution.

The peroxidase (POD) activity was determined based on the method reported by Cao et al. [20] with slight modifications. Each 1 mL juice was diluted by 1–5 times and then mixed with 2 mL 1.0% (*v*/*v*) guaiacol (0.2 M, pH 6.5 phosphate buffer) and 0.2 mL 1.5% hydrogen peroxide as the enzymatic reaction solution.

The absorbance at 420 nm and 470 nm of PPO and POD enzymatic reaction solutions were immediately recorded by a spectrophotometer (Ultrospec 7000, Biochrom LTD, Cambridge, UK) at intervals of 2 s for 4 min. The specific activities of PPO and POD (Abs/min) were calculated according to the slopes of the linear portion of reaction curves, and the residual activities of enzymes were calculated according to Equation (3):

Residual activity of enzymes (%) = 100 × *A*_t_/*A*_0_ (3)

where *A*_t_ and *A*_0_represent the specific activities of treated and untreated samples.

#### 2.4.6. Browning Degree

The browning degree (BD) of juice was determined using the method of Roig et al. [21]. Each 3 mL properly diluted sample was filtered through a 0.45 μm polytetrafluoroethylene syringe filter (Wuxi Huabiao Scientific Instrument Co., Ltd., Wuxi, China). The absorbance of filtrate at 420 nm was measured at 25 ± 1 °C and expressed as BD value.

### 2.5. Statistical Analysis

All data obtained in this study were expressed as mean value ± standard deviation (SD). Analysis of variance (ANOVA) and Pearson correlation analysis were carried out with SPSS 23.0 (IBM Co., New York, NY, USA) using Duncan’s test, and the significance level was *p* = 0.05.

## 3. Results and Discussion

### 3.1. Microbial Growth

**Figure 2:**
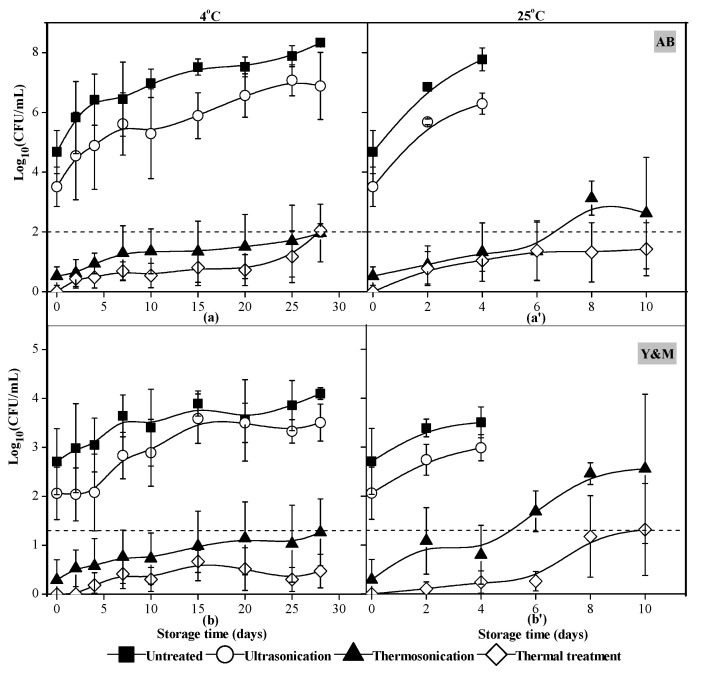
Changes of aerobic bacteria (**a**,**a’**), yeast and molds (**b**,**b’**) in red pitaya juices during storage at 4 °C and 25 °C, as subjected to thermosonication (475 W, 56 °C, 20 min), ultrasonication (475 W, 10 °C, 20 min), and thermal (83 °C, 1.5 min) treatments. Dotted lines were the acceptable limit defined by GB 7101-2015 [23].

TS and thermal treatments effectively decreased the counts of AB and Y&M in clear red pitaya juices to less than 10 CFU/mL, while the US treatment had a negligible effect (Figure 2). Similarly, complete inactivation of total plate count (4.6-log) and enterobacteria (4.2-log) was obtained in green cactus pear juice that was treated by ultrasound waves at 60% amplitude level for 15–25 min with outlet temperature at 58–66 °C and at 80% amplitude level at 71–76 °C [22]. Although both red pitaya and green cactus pear belong to cactus species, a higher initial pH (5.68 vs. 4.90) while a lower initial microbial level (e.g., 4.6-log vs. 4.9-log of total plate count) was found in the above-mentioned study as compared to ours, which may support the better inactivation effects under equal level of TS treatments.

During storage at 4 °C for 28 days and at 25 °C for 10 days, TS- and thermal-treated clear red pitaya juices showed similar trends in AB and Y&M growth, which was much slower as compared to the untreated and US-treated juices. The growth of AB and Y&M in both TS- and thermal-treated juices were mainly due to existing few residual microorganisms. Similar increase trends in naturally occurring microorganisms in fruit and vegetable juices during storage were reported as subjected to TS and thermal processing [12,13,24]. For instance, the microbial counts in carrot juice reduced under the detection limit (<1 CFU/mL) after sonicated at 58 °C for 10 min, but the numbers of mesophiles and Y&M increased to 3.1-log and 4.56-log during storage at 4 °C on day 20 [12]. It should be noticed that the pH value of carrot juice was 6.80 in the above-mentioned study, which provided a reasonable environment for microbial growth in theory. The total mesophilic bacteria and total psychrophilic bacteria in strawberry juice that heated at 90 °C for 60 s and then stored at 5 °C for 10 days increased to 3.20-log and 3.44-log, respectively, but Y&M did not grow [24]. Several non-thermal technologies had been applied to pitaya juice processing. No growth of inoculated *Saccharomyces cerevisiae* in pitaya (*Stenocereus pruinosus*) juice that treated by HHP at 400–600 MPa for 12–16 min and during storage at 4 ± 1 °C up to 15 days was reported [8]. Both *Listeria innocua* and *S. cerevisiae* populations in pitaya (*Stenocereus pruinosus*) juice even decreased to non-detectable levels at 10 days of storage, treated by combining exposure to O_3_ for 7 min and followed by HHP at 316 MPa for 5 min [4]. Besides that, the aerobic mesophiles and Y&M in pitaya juice remained at non-detectable levels as treated by O_3_ for 7 min and followed by HHP at 316 MPa for 5 min, and then stored at 5 ± 2 °C for 30 days, but the number of Y&M abruptly accelerated to 3.37-log on day 35 [4]. These results suggested that a single inoculated strain in pitaya juice would be inactivated more easily than natural microbiota due to the possible presence of resistant strains. Therefore, high levels of TS treatments would be required to eliminate the resistant ones in clear pitaya juice to ensure microbial safety during storage.

Growth of both AB and Y&M at 25 °C were faster than that stored at 4 °C, in all cases. Higher counts of AB and Y&M in untreated and US-treated samples were observed, which led to a fast and obvious increase in the natural microbiota during storage. Therefore, the untreated and US-treated juices showed signs of decay with off-odor after 4 days of storage at 25 °C. These samples were discarded for microbial and chemical analyses once visible signs of decay were observed. The results suggested that TS-treated sample was suitable for consumption after storage at 4 °C for 28 days, as it met the safety standards (<100 CFU/mL) of microbial counts [23].

### 3.2. SSC, pH and Color

**Figure 3:**
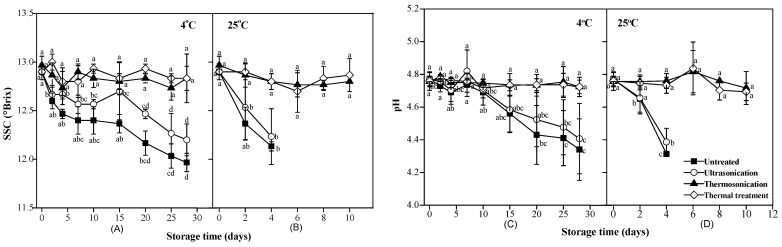
Variance of soluble solid content ((**A**), samples stored at 4 °C; (**B**), samples stored at 25 °C) and pH values ((**C**), samples stored at 4 °C; (**D**), samples stored at 25 °C) in red pitaya juices during storage at 4 °C and 25 °C, as subjected to thermosonication (475 W, 56 °C, 20 min), ultrasonication (475 W, 10 °C, 20 min), and thermal (83 °C, 1.5 min) treatments, a, b, c, d means significant difference of SSC or pH values with respect to storage time (*p* < 0.05).

The SSC and pH values of clear red pitaya juice did not change significantly after treatment by TS (*p* > 0.05), as showed in Figure 3. The results were consistent with previous researches [15]. For instance, similar decrease in pH values of carrot, cactus pear, and orange juice during storage were reported [22,25]. Similar variation trends of SSC and pH were observed in thermal-treated samples.

However, the SSC and pH values of untreated and US-treated samples reduced similarly and significantly (*p* < 0.05). The SSC values reduced to 11.97–12.2 ^o^Brix and 12.13–12.23 ^o^Brix after storage for 28 days at 4 °C and 10 days at 25 °C, respectively. Further analysis revealed a high correlation between the changes in SSC and pH values of untreated and US-treated samples during storage, with a great linear correlation coefficient (*R*^2^_adj_ = 0.98). Like the variation of the microbial counts in clear red pitaya juice, slower changes in SSC and pH values were observed at a lower storage temperature. This phenomenon was considered relating to the metabolism of natural microbiota in untreated and US-treated clear red pitaya juice. It had been reported that acidity increases in juices when microorganisms use sugars as nutrients to produce organic acid, thus increasing acid concentration [26,27]. Soluble sugars, including glucose (75 mg/mL) and fructose (22 mg/mL), make up the majority of SSC in clear red pitaya juice [14]. They provide adequate carbon sources for lots of microorganisms. Similarly, it has been reported that the microbes metabolized sugar to produce organic acids, which leads to a decrease in pH and total sugars levels in orange juice [13]. Different results have been reported in processing carrot juice with TS; the pH value of carrot juice treated by TS at 58 °C presented a significant reduction after 20 days of storage (*p* < 0.05), although the sample did not show obvious spoilage [12]. They explained that the changes in pH after sonication were related to the generation of new chemical compounds and enzymatic changes in the media.

**Figure 4:**
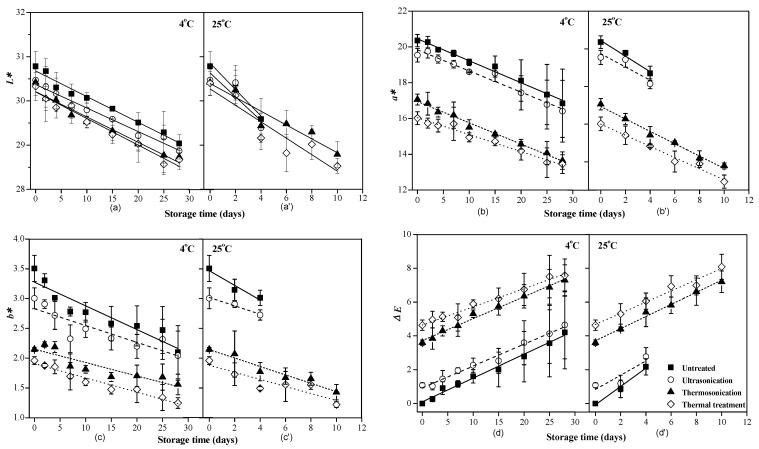
Variance in color of red pitaya juices during storage at 4 °C and 25 °C, as subjected to thermosonication (475 W, 56 °C, 20 min), ultrasonication (475 W, 10 °C, 20 min), and thermal (83 °C, 1.5 min) treatments. (**a**,**a’**) Description of L* values during storage; (**b**,**b’**) Description of a* values during storage; (**c**,**c’**) Description of b* values during storage; (**d**,**d’**) Description of Δ*E* values during storage. Dotted lines are fitting curves with the linear model.

The color indices of clear red pitaya juices during storage are shown in Figure 4. The L*, a* and b* values of untreated juice were 30.78, 20.36, and 3.51, respectively. As subjected to US, TS, and thermal treatments, these color indices decreased, and the Δ*E* values were 1.08, 3.59, and 4.63, respectively. TS treatment induced decrease in L* value, that is related to the changes of a* and b* values, and also to the browning substances generated from the Maillard reaction [14]. The decomposition of betacyanins directly resulted in the reduction in a* value. It might be explained as such: The nature of direct reaction of ultrasonic waves and lights with free radicals or secondary oxidants led to decomposition of betacyanins during storage in transparent glass bottles [28]. In addition, microbial metabolism generates substances like acid, alcohol, and gas might interfere with the stability of betacyanins and the color appearance of juice. Similar change of color indices of TS-treated juices during storage has been reported [13,25]. For instance, the L*, a* and b* values of TS-treated (56 °C for 6 min or 60 °C for 5 min) carrot juice decreased during storage at 4 °C for 21 days [25]. With regard to pitaya juice processing, a combination of O_3_ and HHP at 316 MPa for 5 min caused reduction in L* value and its chroma, which was accelerated during the 30 days storage and eventually resulted in Δ*E* of 3.8 units [4]. According to our previous study, a Δ*E* of 5.0 units is a visible color difference threshold for clear red pitaya juice [14]. Invisible color change of TS-treated clear red pitaya juice was retained for 10 days at 4 °C, which was longer than thermal-treated samples for 2 days (Figure 4). The results indicated a better color retention ability through TS than thermal treatment.

### 3.3. Betacyanins Content

**Figure 5:**
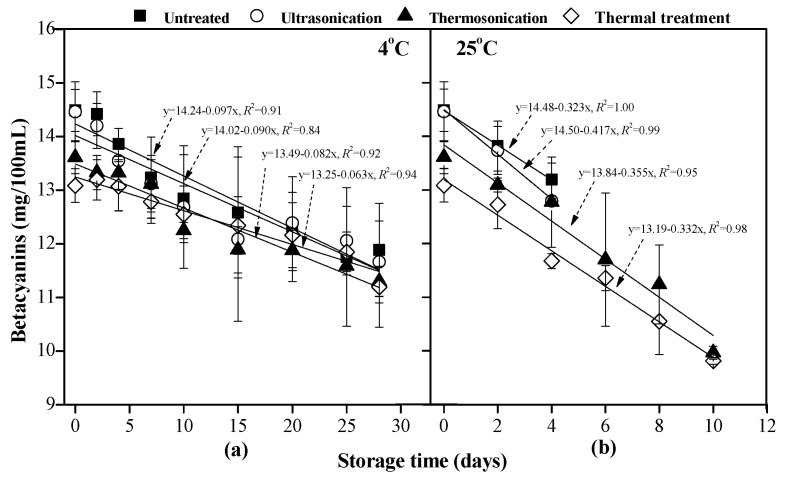
Change of the contents of betacyanins in red pitaya juices during storage at 4 °C (**a**) and 25 °C (**b**), as subjected to thermosonication (475 W, 56 °C, 20 min), ultrasonication (475 W, 10 °C, 20 min), and thermal (83 °C, 1.5 min) treatments. Dotted lines are fitting curves with the linear model.

The initial content of betacyanins in clear red pitaya juice was 14.49 mg/100 mL; it decreased to 13.09–13.61 mg/100 mL after being treated by TS and thermal processing (*p* < 0.05), as shown in Figure 5. Under the storage conditions of 4 °C and 25 °C, the betacyanins content decreased linearly with the storage time, indicating loss in betacyanins during storage. The variation in betacyanins content at 25 °C was 3.3–5.3 times higher than that at 4 °C (based on the *k* values of the linear fitted line), indicating a faster decomposition rate of betacyanins when stored at higher temperature. At the end of the storage at 4 °C, 83.0% betacyanins were retained in TS-treated samples.

Betacyanins undergo C15-isomerization, deglycosylation, dehydrogenation, hydrolysis, and decarboxylation during processing [29], leading to changes in the color of resulting food products. With regard to TS processing, the degradation and isomerization of betanin and phyllocactin are involved in the decomposition of betacyanins primarily [14]. In this study, significant decomposition of betacyanins in all samples were observed during storage (*p* < 0.05), which was slower at 4 °C than 25 °C. Similarly, after 8-week storage, the amount of betacyanins in red pitaya juice at 4 °C was more than twice of it at 25 °C [30]. According to observed results, automatic degradation of betacyanins occurred during storage in the present of lights, and this reaction was accelerated at a higher temperature. According to [3], partial regeneration of betanin occurs after short-term heating, though this phenomenon did not present in this study. In the present study, partial regeneration of betacyanins in the thermal-treated sample during storage might exist, as the lowest content of betacyanins presented in the thermal-treated sample. However, its retention rate (85.6% at 4 °C, as compared to that on day 0) was the highest one among all cases, suggesting that retained betacyanins in the thermally treated pitaya juices had a relatively high stability. To the best of our knowledge, it is the first time to report the decomposition dynamics of betacyanins in red pitaya juice after treatment by TS processing during storage. Future study can investigate its decomposition pathway during storage in order to minimize color decay of juices.

### 3.4. PPO and POD Activities

Endogenous enzymes such as PPO and POD are generally considered to ultimately lead to formation of dark brown polymers with quinoan properties, resulting in browning of fruit and vegetable products. Several studies have reported that TS inactivates PPO and POD [9,31]. For example, 93.85% PPO and 91% POD in apple juice are inactivated by TS at 525 W and 60 °C within 10 min [9]. Besides that, enzymatic oxidation of betanidin and betanin by horseradish peroxidase were reported by Wybraniec and Michałowski [32]. Possible disturbance of betalain stability by degrading endogenous enzymes such as PPO and POD has been proposed by Khan [33]. Herein, the influence of endogenous PPO and POD residues on betacyanins and resulting color change of clear red pitaya juice should be considered.

**Figure 6:**
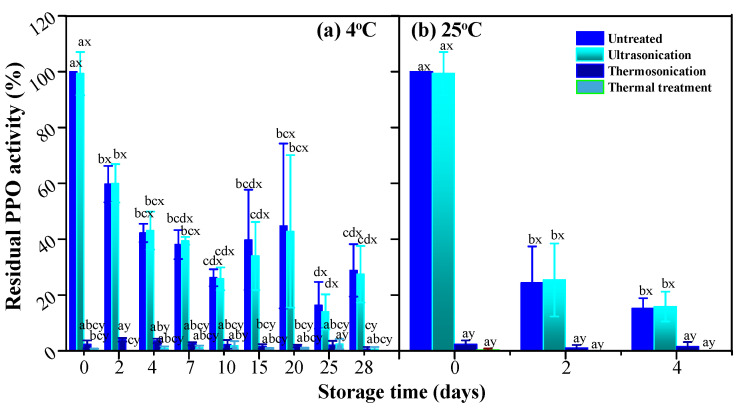
The residual activity of PPO in red pitaya juices during storage at 4 °C (**a**) and 25 °C (**b**), as subjected to thermosonication (475 W, 56 °C, 20 min), ultrasonication (475 W, 10 °C, 20 min), and thermal (83 °C, 1.5 min) treatments. a, b, c, d means significant difference with respect to storage time (*p* < 0.05); x, y, z, i means significant difference with respect to various treatments (*p* < 0.05).

**Figure 7:**
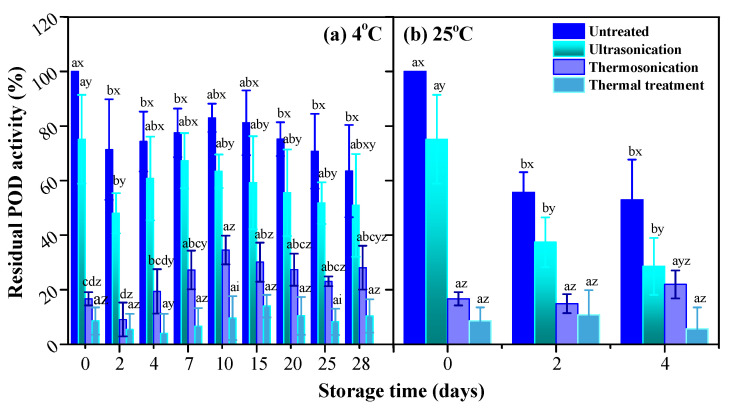
The residual activity of POD in red pitaya juices during storage at 4 °C (**a**) and 25 °C (**b**), as subjected to thermosonication (475 W, 56 °C, 20 min), ultrasonication (475 W, 10 °C, 20 min), and thermal (83 °C, 1.5 min) treatments. a, b, c, d means significant difference with respect to storage time (*p* < 0.05); x, y, z, i means significant difference with respect to various treatments (*p* < 0.05).

As compared with untreated samples, PPO (Figure 6) and POD (Figure 7) activities reduced drastically after exposure to TS and thermal treatments. There were 2.3% and 16.7% residual enzyme activities of PPO and POD retained in TS-treated juice sample, and that did not change significantly (*p* > 0.05) during storage at 4 °C and 25 °C for 28 days and 10 days. With regard to US treatment, negligible inactivation of PPO (99.33% residual activity) and 24.9% reduction in POD activity were observed. These results indicated heat sensitivity of PPO and inactivation of POD by sonication and/or heat. A synergistic effect between thermal process and ultrasonic waves in the form of TS facilitated and resulted in enzymes deactivation. Moreover, limited residual enzyme activities of TS-treated samples did not contribute to enzymatic browning of juice during the whole experimental period, nor influence betacaynins retention.

### 3.5. BD Value

Change in BD values of clear red pitaya juice samples during storage are showed in Figure S1. A significant increase in BD values of red pitaya juices were observed as treated by TS and thermal processing (*p* < 0.05), with the order of thermal-processed juice > TS-processed juice. The BD values did not vary during storage both at 4 °C or 25 °C (*p* > 0.05). Non-enzymatic reactions between amino acids and carbohydrates caused by food processing persist during storage [34], but this reaction was not the main cause of the darkening of clear red pitaya juice.

### 3.6. Pearson Correlation Analysis

To analyze the multiple correlation of microbial and quality indices of TS- and thermal-treated clear red pitaya juices, a Pearson correlation analysis was conducted and is shown in Table 1. Δ*E* was significantly and negatively correlated with betacyanins content (−0.91–−0.98) in both processes, and betacyanins content was significantly positively correlated with a* (0.91–0.97). These indicated that the decomposition of betacyanins in clear red pitaya juice resulted in decrease of a* value and which led to the acceleration of color change (increase of Δ*E*) ultimately during storage. Besides that, Δ*E* of TS-treated samples presented a positive correlation with microbial counts (0.96 for AB; 0.85 for Y&M), and Δ*E* of thermal-treated samples presented positive but weaker correlation with microbial counts (0.85 for AB; 0.67 for Y&M with *p* < 0.05). It indicated that, even as low as less than 10 CFU/mL residual native microbiota, the followed growth of these microbiota during storage influenced the quality of juice. Effective control of spoilage and pathogenic microbiota is a key aspect of microbial safety and food quality, especially during storage. Conversely, color or its change can serve as an indicator of microbial quality during storage of fruit and vegetable juices, which was consistent with a previous study [35]. The betacyanins contents of TS- and thermal-treated samples had a significant negative correlation with AB (−0.90–−0.92) and Y&M (−0.61–−0.83), indicating that effective control of microbiota would facilitate retention of betacyanins. Residual PPO and POD did not have significant correlation with the color properties of thermal-treated juices, even though they had significant correlation with Δ*E* and a* values of TS-treated samples. BD had a significant positive correlation with color indices (>0.73) in both TS- and thermal-treated samples.

## 4. Conclusions

Results of the present study demonstrated that TS can be used as an alternative technology of thermal pasteurization in red pitaya juice processing, especially for microbial safety and physico-chemical quality control. The quality and stability of TS-treated red pitaya juices was equal to that treated by short time thermal processing at 83 °C. Though slight growth of native microbiota in TS-treated juices was observed during storage for 28 days at 4 °C, they were below the acceptable limits defined by GB 7101-2015. Enzymatic browning and non-enzymatic browning were not the main reasons of color decay during storage, while the decomposition of betacyanins and the metabolism of residual native microbiota contributed to color decay. Slight increases in temperature might be better in controlling native microbiota in juice processing using TS technology, which may be conducive to food safety and quality control during storage. In that case, a shorter treatment time could be used to facilitate industrial application of TS. Further research can study the optimal treatment conditions for pilot or scaled-up juice processing in the future. 

## Figures and Tables

**Table 1:** Pearson correlation of microbial and quality parameters of thermosonication- (with gradient pink background) and thermal-treated clear red pitaya juice during storage.

	Δ*E*	L*	a*	b*	Betacyanins	pH	SSC	PPO	POD	BD	AB	Y&M
**Δ*E***	1	−0.98 **	−1.00 **	−0.96 **	−0.91 **	−0.73 **	−0.47	0.51	0.38	0.75 **	0.85 **	0.67 *
L*	−0.98 **	1	0.97 **	0.96 **	0.91 **	0.73 **	0.54	−0.52	−0.27	−0.73 **	−0.83 **	−0.71 **
a*	−1.00 **	0.97 **	1	0.96 **	0.91 **	0.73 **	0.45	−0.50	−0.38	−0.75 **	−0.85 **	−0.65 *
b*	−0.94 **	0.93 **	0.93 **	1	0.94 **	0.81 **	0.55	−0.45	−0.41	−0.78 **	−0.89 **	−0.77 **
**Betacyanins**	−0.98 **	0.95 **	0.97 **	0.92 **	1	0.80 **	0.56	−0.25	−0.31	−0.67 *	−0.90 **	−0.61 *
**pH**	−0.75 **	0.68 *	0.75 **	0.76 **	0.73 **	1	0.50	−0.38	−0.55	−0.49	−0.64 *	−0.66 *
**SSC**	−0.69 *	0.71 *	0.69 *	0.53	0.65 *	0.43	1	−0.38	−0.05	−0.33	−0.54	−0.58 *
**PPO**	−0.59 *	0.45	0.60 *	0.68 *	0.57	0.65 *	0.05	1	0.10	0.42	0.21	0.41
**POD**	0.65 *	−0.66 *	−0.64 *	−0.79 **	−0.69 *	−0.76 **	−0.45	−0.43	1	0.16	0.23	0.55
**BD**	0.77 **	−0.81 **	−0.77 **	−0.80 **	−0.67 *	−0.43	−0.53	−0.46	0.44	1	0.73 **	0.61 *
**AB**	0.96 **	−0.96 **	−0.96 **	−0.94 **	−0.92 **	−0.76 **	−0.70 *	−0.56	0.74 **	0.83 **	1	0.59 *
**Y&M**	0.85 **	−0.79 **	−0.86 **	−0.79 **	−0.83 **	−0.65 *	−0.57	−0.68 *	0.49	0.69 *	0.85 **	1

* Significant at 5% (*p* < 0.05), ** Significant at 1% (*p* < 0.01).

## Data Availability

The data presented in this study are available on request from the corresponding author.

## Supplementary Materials

The following are available online at https://www.mdpi.com/article/10.3390/foods10051041/s1, Figure S1: The change of browning degree (A420 nm) of red pitaya juices during storage at 4 °C and 25 °C, as subjected to ultrasonication (475 W, 10 °C, 20 min), thermosonication (475 W, 56 °C, 20 min), and thermal treatment (83 °C, 1.5 min).
