# Supplementary material for: Application of Thermosonication in Red Pitaya Juice Processing: Impacts on Native Microbiota and Quality Properties during Storage
Source: Foods. 2021 May 10;10(5):1041. doi: 10.3390/foods10051041 (PMC8151109; doi:10.3390/foods10051041)
Supplement: Supplementary file 1 [file foods-10-01041-s001.zip › foods-1189811-supplementary.pdf]

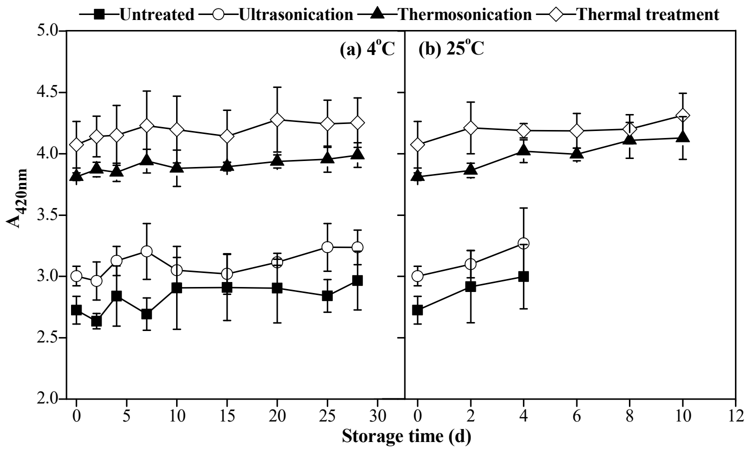

Figure S1. The change of browning degree (A_{420nm}) of red pitaya juices during storage at 4 °C and 25 °C, as subjected to ultrasonication (475 W, 10 °C, 20 min), thermosonication (475 W, 56 °C, 20 min), and thermal treatment (83 °C, 1.5 min).
